# Considerations for conducting and reporting digitally supported cognitive interviews with children and adults

**DOI:** 10.1186/s41687-021-00371-5

**Published:** 2021-12-18

**Authors:** Abigail Fry, Sandra A. Mitchell, Lori Wiener

**Affiliations:** 1 Pediatric Oncology Branch, National Cancer Institute, Center for Cancer Research, National Institutes of Health, Bethesda, MD USA; 2 Outcomes Research Branch, Healthcare Delivery Research Program, Division of Cancer Control and Population Sciences, National Cancer Institute, National Institutes of Health, 8909 Medical Center Drive, 3E-448, Rockville, MD 20850 USA

**Keywords:** Qualitative interviewing, Cognitive debriefing, Instrument development, Digitally supported research methods, Pediatrics, Patient-reported outcomes, Remote qualitative research

## Abstract

**Background:**

Cognitive interviewing is a well-established qualitative method used to develop and refine PRO measures. A range of digital technologies including phone, web conferencing, and electronic survey platforms can be leveraged to support the conduct of cognitive interviewing in both children and adults. These technologies offer a potential solution to enrolling underrepresented populations, including those with rare conditions, functional limitations and geographic or socioeconomic barriers. In the aftermath of the COVID-19 pandemic, the use of digital technologies for qualitative interviewing will remain essential. However, there is limited guidance about adapting cognitive interviewing procedures to allow for remote data capture, especially with children.

**Methods:**

Synthesizing the literature and our research experiences during the COVID-19 pandemic, we examine considerations for implementing digitally supported cognitive interviews with children, adolescents, and adults. We offer recommendations to optimize data quality and empirical rigor and illustrate the application of these recommendations in an ongoing cognitive interviewing study to develop and refine a new pediatric PRO measure.

**Results:**

Good research practices must address participant and researcher preparation for study-related procedures and should anticipate and pre-emptively manage technological barriers. Field notes should detail interview context, audio/video cues, and any impact of technological difficulties on data quality. The approaches we recommend have been tested in an ongoing cognitive interviewing study that is enrolling children/adolescents with cGVHD ages 5–17 and their caregivers [NCT 04044365]. The combined use of telephone and videoconferencing to conduct cognitive interviews remotely is feasible and acceptable and yields meaningful data to improve the content validity of our new PRO measure of cGVHD symptom bother.

**Conclusion:**

Digitally supported cognitive interviewing procedures will be increasingly employed. Remote data collection can accelerate accrual, particularly in multi-site studies, and may allow for interviewer personnel and data management to be centralized within a coordinating center, thus conserving resources. Research is needed to further test and refine techniques for remote cognitive interviewing, particularly in traditionally underrepresented populations, including children and non-English speakers. Expansion of international standards to address digitally supported remote qualitative data capture appears warranted.

## Background

Patient-reported outcome (PRO) measures are essential tools to capture patient-centered endpoints in both observational research and clinical trials. Cognitive interviewing is a well-established qualitative method used to develop and refine PRO instruments. However, it can be challenging to sample geographically and socio-economically diverse individuals, children, and those with rare conditions or physical/functional limitations for qualitative research, including cognitive interviews [1]. Remote methods (which include telephone, web conferencing, and other social media and messaging platforms) offer a potential solution to enrolling these underrepresented populations [2]. Remote methods also allow for interviewer personnel and data management to be centralized within the study coordinating center. That centralization is particularly useful for multi-site research as it avoids the need to recruit, train, and supervise interviewers in multiple study sites. Centralization may thus serve to enhance efficiency, conserve resources, and improve methodologic quality. Innovative methods, including successfully adapting technology to address these challenges and facilitate the conduct of rigorous qualitative research, are warranted.

The use of remote methods to collect interviewer-administered survey data has been well described [3]. Similarly, there is a growing literature on leveraging social media, texts, blogs, chats, and instant messages to capture qualitative data online [4]. A small methods literature supports the feasibility, acceptability, and meaningfulness of remote qualitative interviews as an alternative to focus groups and individual interviews conducted in-person [5, 6]. However, the adaptation of traditional face-to-face cognitive interviewing principles to the remote environment, particularly with children and adolescents, has not been well described. To address this knowledge gap, this paper summarizes considerations and strategies for designing, performing, and reporting cognitive interviews conducted remotely using digital technologies. We illustrate the application of good research practices in an ongoing study to develop a new pediatric PRO measure. Lessons learned and key considerations to strengthen the empirical rigor of remote cognitive interviews are discussed.

## Methods

Cognitive interviewing aims to evaluate and iteratively refine a PRO measure by gathering direct input from respondents about item content, comprehension, ease of response and format [7]. Cognitive interviewing addresses several areas that often need improvement during PRO instrument development, including clarity and comprehension, cognitive recall burden, response choices, ease of judgement, and questionnaire formatting and layout [8, 9]. The overall purpose of cognitive interviewing is to minimize measurement error by determining that research participants interpret question concepts as intended and can provide accurate responses.

While there can be variation in the structure and sequence of cognitive interviewing techniques, the basic structure involves two parts. In the first part, survey questions are administered to the respondent. This is followed by a semi-structured debriefing interview where the respondent is encouraged to reflect and provide feedback on the comprehension of each survey question, the clarity of interpretation, and their ease in selecting a response.

Traditional cognitive interviewing methods require adaptation to the remote environment, due to the active, structured, and highly reciprocal process that occurs between study participant and researcher. The need to administer survey questions prior to debriefing can make remote cognitive interviews less amenable to being exclusively telephone-based. As such cognitive interviews are greatly aided by the inclusion of the visual component that videoconferencing technologies offer.

## Results

We illustrate the principles underlying the implementation of digitally supported cognitive interviewing in a multi-site study to develop, refine and test a new symptom scale for children and adolescents. The study is enrolling a sample of pediatric transplant survivors ages 5–17 with chronic graft-versus-host-disease (cGVHD) and their parents/caregivers (NCT04044365). The rare patient population, geographic dispersion, and the need to centralize methodologic expertise in conducting cognitive interviews with children motivated our use of remote interviewing methods. In designing our approach, we also had to accommodate several contextual challenges. These included: (1) the intricacies of interviewing children at different developmental stages, (2) inclusion of a child-parent dyadic interview component, (3) the requirement to balance participant burden with the need to debrief on a large number of PRO items, and (4) study participants’ prominent illness severity.

### Implementation of remote cognitive interviewing methods

In this study, we employ both synchronous and asynchronous digital approaches to support remote data collection. To fulfill the first part of the cognitive interview, the child completes the symptom scale facilitated by a combination of screensharing on the videoconferencing platform and telephone for audio. The child views each PRO item on their computer screen and provides their verbal response, while the interviewer notes any difficulties such as hesitancy or indicators of confusion (such as changing answers). During the second part of the cognitive interview, to facilitate recall and engagement, screen sharing is used to revisit the items that the child experienced as problematic. Child-parent dyadic debriefing is also incorporated to identify and explore areas of miscomprehension that may be indicated by discordant ratings between child and parent. To accomplish this, the parent completes the caregiver proxy survey in advance of the interview. Completing this asynchronously both conserves time during the child interview and ensures that any discordant ratings are available so that the interviewer can return to these items when child-parent are jointly debriefed.

**Fig. 1:**
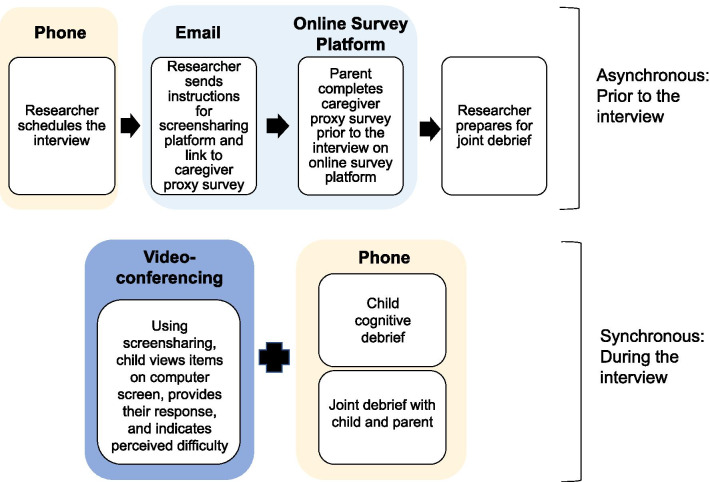
Flow of data collection

Figure 1 depicts the flow of data collection and integration of technological approaches. The three technologies are complementary and synergistic and were chosen with intentionality to address the study aims. There are a number of videoconferencing software systems from which to choose. Features that were important in our study included compliance with Health Insurance Portability and Accountability Act (HIPAA) guidelines, ease of screensharing, and the simplicity of a single-click access without the participant requiring an account or a password protected log-in. We also considered the user experiences of both interviewers and study participants, as gathered during pretesting. Inclusion of the telephone component reduces some of the technical complexity for both child and parent and facilitates participation by respondents with limited broadband access. Since video may not be consistently employed throughout the interview, verbal cues such as silence, which may indicate that the child is becoming frustrated, fatigued, or experiencing distress or disengagement, are closely monitored. Screensharing offers a visual component that encourages child engagement. The material presented during screensharing incorporates features such as embedded animation and markers to track progress. These features promote rapport and allow the child to feel a sense of control over the interview process. As with all cognitive interviewing, to mitigate social desirability biases, our interview guide reinforces that there are no right or wrong answers. The interviewer avoids evaluative language (e.g., “good answer”), using encouraging language instead (e.g., “this is very helpful information”). We have found that this combination of synchronous and asynchronous remote strategies ensures that both the child report and the parent perspective are captured in a fully independent manner.

Our remote cognitive interviewing procedures have enabled participant recruitment at more than ten centers while allowing for interviewers with unique expertise in interviewing children to be centralized at the coordinating center. Digital technologies have successfully facilitated child engagement during the cognitive interview, even among children as young as 5–7 years. Our experiences support the feasibility and acceptability of conducting digitally supported cognitive interviews, and our findings have offered meaningful insights about the comprehension, clarity, and ease of response of this new pediatric symptom scale.

### Considerations to strengthen empirical rigor

Migrating cognitive debriefing interviews to a fully remote methodology requires that several considerations be addressed (see Table 1). Participant access to a computer and broadband internet (mediated by geographic factors and socioeconomic status), data security through online platforms, rapport, participant fatigue and engagement (mediated by age and time looking at screens), digital literacy, digital failures and resultant data loss should all be considered as potential limitations to be mitigated [10]. Having a second researcher present during the remote interview offers several advantages. These include sharing of technical tasks, providing the primary interviewer with suggestions for additional probing, and helping to manage data collection [10, 11].

**Table 1:**
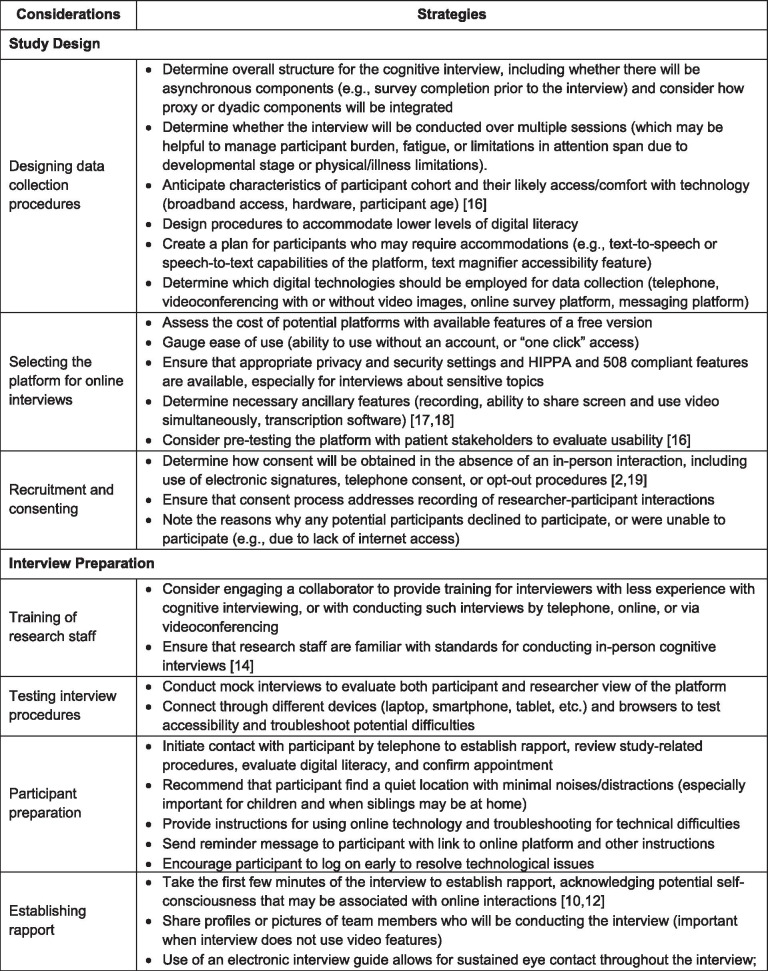

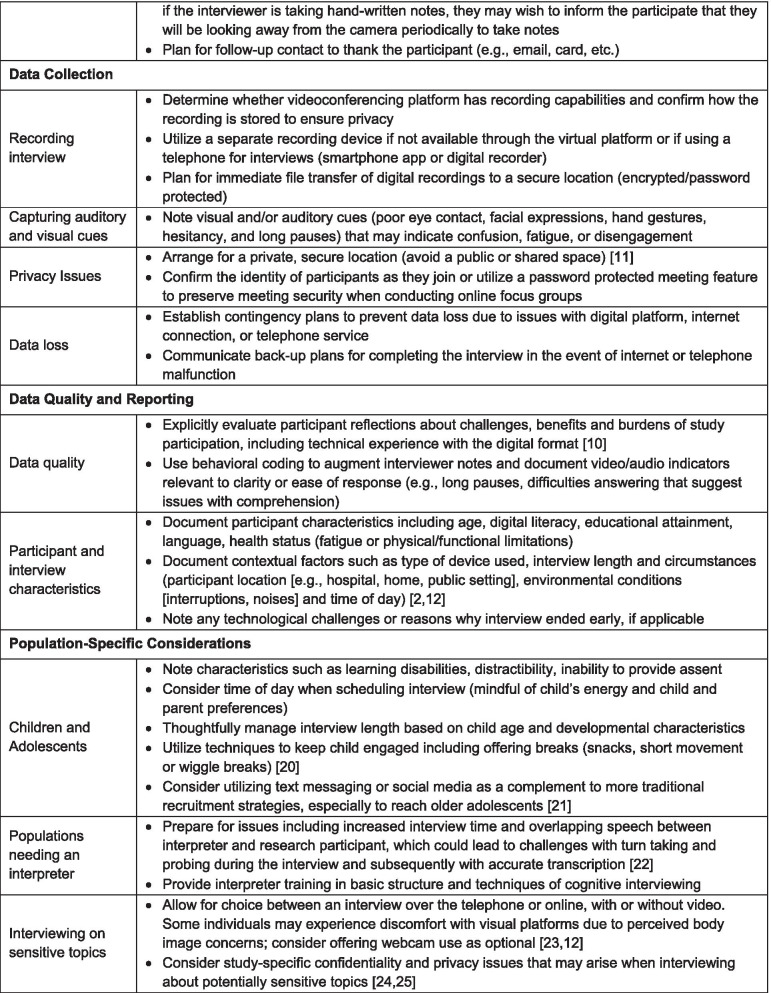
Considerations for conducting digitally supported cognitive interviews

A semi-structured interview guide is an essential component of all rigorously conducted cognitive interviews; it helps to ensure that the process is systematic and well-documented [7, 8]. This standardization is also critically important since technological challenges and procedural interruptions may occur more frequently with remote cognitive interviewing and can be distracting for both interviewer and participant. Procedural interruptions include environmental distractions, interviewee reluctance to speak freely due to the presence of family members, intrusions resulting from day-to-day activities in the home, and difficulties with phone or internet connectivity and audio/video quality. The interview guide, which may be electronic, or paper based, serves to prompt the interviewer to document the various forms of cognitive difficulties (e.g., clarity, comprehension, ease of response) that occur during the interview, along with relevant visual or auditory indicators of these difficulties (e.g., hesitation) [2, 11, 12]. The interview guide should also offer structured fields to record environmental conditions, participant engagement, technological aspects (e.g., type of device(s) utilized by the participant, use of video versus audio only), and any problems or difficulties encountered during the interview. To facilitate interpretation of results, the published report should summarize the technological and contextual interview, and detail any associated limitations in sampling, such as participant exclusion or withdrawal. Interviewer proficiencies that strengthen the empirical rigor of digitally supported cognitive interviews include strong knowledge of cognitive interviewing principles, a capacity for agile navigation within and between digital platforms, and responsiveness to unique participant challenges including technical difficulties [10].

## Discussion

The COVID-19 pandemic has stimulated opportunities to maximally leverage digital technologies to facilitate research participation and support data collection, and it is anticipated that these approaches will continue to be relevant [12]. Synthesizing participant experiences with digitally supported cognitive interviews across studies could produce new insights into how best to adapt our methods for specific study populations and topic areas. Methodologic questions for future research and policy development include: Who participates in this research and who declines, and for what reasons? Does study participation, respondent engagement, and data quality vary by age, disease type, digital literacy, educational attainment, language literacy/acculturation, or other participant characteristics? What are the best practices for obtaining electronic consent? To what extent might digitally supported cognitive interview methods introduce bias, and what strategies are effective in limiting potential sources of bias? Can the cognitive interview data that is captured remotely, and the data collected during an in-person interview be pooled for analysis? Methodologic standards and best practices for cognitive interviewing [13, 14] should, in future iterations, address considerations for conducting digitally supported cognitive interviews. Lastly, there is a need to test, refine and scale recently described technological innovations that support inclusion of study participants who do not have access to computer hardware or internet connectivity [15].

## Conclusion

This paper has highlighted considerations and illustrated strategies for adapting cognitive interviewing methods to a remote environment. As technology evolves, opportunities exist to extend the application of these approaches and refine their use in diverse research contexts. Remote cognitive interviewing methods have broad applicability for PRO researchers, particularly those studying rare conditions and recruiting populations who have traditionally been underrepresented in research. This approach also allows for interviewers and data management to be centralized; this may be particularly useful in enhancing efficiency in multi-site studies. Our experiences demonstrate that digital technologies can be successfully implemented to support remote conduct of cognitive interviews, including with children and adolescents, while preserving the methodologic principles that ensure optimal data quality and empirical rigor.

## Data Availability

Not applicable.

## Abbreviations

PRO: Patient-reported outcome
cGVHD: Chronic graft-versus-host-disease
